# Hydrothermal Ageing of Glass Fibre Reinforced Vinyl Ester Composites: A Review

**DOI:** 10.3390/polym15040835

**Published:** 2023-02-08

**Authors:** James Thomason, Georgios Xypolias

**Affiliations:** Department of Mechanical and Aerospace Engineering, University of Strathclyde, 75 Montrose Street, Glasgow G1 1XJ, UK

**Keywords:** vinyl ester, glass fibre, composite, mechanical properties, environmental ageing, hydrothermal ageing, fibre-matrix interface, voids

## Abstract

The use of glass fibre-reinforced polymer (GFRP) composites in load-carrying constructions has significantly increased over the last few decades. Such GFRP composite structures may undergo significant changes in performance as a consequence of long-term environmental exposure. Vinyl ester (VE) resins are a class of thermosetting polymers increasingly being used in such structural composites. This increasing use of VE-based GFRPs in such applications has led to an increasing need to better understand the consequences of long-term environmental exposure on their performance. The reliable validation of the environmental durability of new VE-based GFRPs can be a time- and resource-consuming process involving costly testing programs. Accelerated hydrothermal ageing is often used in these investigations. This paper reviews the relevant literature on the hydrothermal ageing of vinyl ester-based GFRP with special attention to the fundamental background of moisture-induced ageing of GFRP, the important role of voids, and the fibre-matrix interface, on composite mechanical performance.

## 1. Introduction

The use of glass fibre-reinforced polymer (GFRP) composites in large outdoor infrastructure applications has increased significantly over recent times. Over their lifetime these GFRP composites can exhibit significant changes in performance due to the long-term environmental exposure typical for large load-carrying constructions found in marine structures, transport vehicles, wind turbines and civil infrastructure [1]. It is known that this may reduce the durability of the materials and eventually trigger irreversible degradation mechanisms, which will affect their properties. The increasing use of polymer composites in such applications has led to an increasing awareness of the need to better understand the consequences of long-term environmental exposure on the performance of these composites.

Vinyl ester resins are widely used in structural components in a broad range of applications in the marine, offshore, and civil infrastructure sectors. Although a typical vinyl ester (VE) system may possess lower mechanical properties compared to a high-performance epoxy system, its fast fabrication cycle times, ease of processability, low cost, and adequate mechanical performance levels, often make it an attractive alternative [2,3,4]. Nevertheless, the understanding of the long-term performance of VE-based GFRP is still incomplete and the available literature on the subject is much more limited than that for epoxy-based GFRP. Furthermore, it is well known that the stress transfer capability of the fibre-matrix interface region plays a crucial role in the optimization and long-term durability of GFRP performance [5,6,7,8]. In fact, the condition of the interface can define the reliability and therefore the durability of composite materials or structures during their service life. Although the integrity and properties of the interface region may be particularly sensitive to hydrothermal ageing, most research to date has been focused on the moisture uptake kinetics and failure of bulk composites. Knowledge of the effectiveness of studying interface performance in wet environments as a means of accelerated ageing and how to accurately determine the hydrothermal response of a bulk laminate is still somewhat limited. This paper presents a review of the relevant literature on the hydrothermal ageing of vinyl ester-based GFRP with special attention to the fundamental background of moisture-induced ageing of GFRP and its effects on the fibre-matrix interface and composite mechanical performance.

## 2. Introduction to Ageing

### 2.1. Physical and Chemical Ageing

The process of a change in properties of a material or structure as a function of time is loosely referred to as “ageing”. Depending on the type of degradation mechanisms operating, ageing can be classified into two main categories: “physical” and “chemical”. The rate and degree of degradation throughout ageing are uniquely defined by material type, environmental and mechanical loading, and its duration relative to the expected durability limit [7]. Physical ageing refers to the exposure of materials or components to environmental conditions that do not result in chemical changes in their structure and have reversible effects. In polymer materials, physical ageing is associated with free volume evolution and the change in properties relative to an equilibrium state. For instance, a common physical ageing agent in thermosetting resins is moisture-induced plasticisation caused by moisture diffusion into the polymer network. Moisture-induced plasticisation is known to cause measurable changes in the mechanical properties, as well as glass transition temperature (Tg) reduction of polymeric materials, which nonetheless can be reversed [7,8,9].

The difference between “chemical” and “physical” ageing is primarily defined by the reversibility of the effect, i.e., the ability of the material to regain its properties after it has been exposed to a humid environment. Three of the most important chemical ageing mechanisms are: thermo-oxidation, thermal-induced hydrolytic degradation, and moisture-induced hydrolytic degradation. The latter two effects are reviewed here in more detail since hydrothermal ageing is the primary focus of this study. “Chemical”, moisture-induced ageing is associated with irreversible degradation and changes in the polymer chain/network, mainly occurring through the mechanisms of hydrolysis, chain scission, or even cross-linking for incompletely cured polymeric materials [10,11].

Chemical ageing is more probable when degradation mechanisms, such as moisture and heat or moisture and mechanical loads, act simultaneously. For instance, for a thermoset composite exposed to a humid environment, a temperature increase will accelerate degradation and may cause chemical irreversible changes to the material [10,11,12,13,14]. Similarly, the presence of mechanical loads and residual stresses in a polymeric material may increase the probability of bond rupture and promote chain scission. Chemical ageing can change the properties of polymeric materials and therefore change their diffusion kinetics [7,12].

### 2.2. Ageing Degradation Mechanisms and Accelerated Ageing

The degradation mechanisms acting during the environmental ageing of composites act mainly on the polymeric matrix and the fibre-matrix interface [5,7,13,14]. The main degradation agents acting during environmental ageing include [12]:

- Thermal—high/low temperatures and thermal cycling.
- Moisture—hydrothermal environments, high humidity, water immersion.
- Weathering—UV light exposure combined with rain and/or sand erosion.
- Chemicals—acidic and alkaline environments, solvents and oxygen.
- Irradiation—UV or high-energy radiation.
- Biological—micro-organisms.
- Mechanical degradation (cracking, delamination, voids, fibre fracture).
- Static fatigue/creep rupture.

These mechanisms can act alone or in combination. Combined action can have severe effects on the performance of polymeric materials and may result in complex reactions and irreversible degradation [12]. 

The performance and integrity of in-service materials or structures undergoing natural ageing—exposed to real-life ageing conditions—can be assessed by condition monitoring through non-destructive testing (NDT) or by the physical examination of material components taken out of service. However, in-service monitoring can only provide information on the current state of components and not on the serviceable lifespan of the material or component. Laboratory investigations of environmental ageing under “normal” conditions may have to run for many years and such timescales can be beyond the duration of many academic studies [5,8,13,14]. Therefore, accelerated-ageing studies may provide a validation of the future performance and durability of composite materials and components [7,12]. The basic concept of accelerated ageing is to increase the degradation conditions, such as temperature or humidity, to a higher level than that experienced in real-life operating environments. A material that is subjected to accelerated ageing may therefore reach the same end-state as that in the real-life operating environment in less time. Another common way to accelerate ageing is to reduce the thickness or size of the test specimens [7,12]. 

Accelerated ageing is often employed to assess the life expectancy of new materials used in long-term or critical applications, where the knowledge of their potential failure mechanisms remains limited. The major limitation concerning accelerated ageing is that the increased degradation rates may alter the effect of the degradation agents when compared to real-life operating conditions and weaken the validity of any predictions. Therefore, accelerated ageing results should be coupled with analytical methods to accurately predict future performance and durability. For instance, multiphysics approaches are usually employed to correlate physical ageing with the diffusion of moisture through polymeric materials and its effect on their mechanical performance [7,12]. Additionally, spectroscopic methods such as Fourier-transform infrared spectroscopy (FTIR) are often coupled with gravimetric measurements for quantifying the moisture gain of polymer composites [14].

In general, material testing is associated with high costs, involving many material-related disciplines and a variety of laboratory equipment. It is apparent that, although long-term, real-life testing can fully assess the durability and life expectancy of materials, accelerated laboratory-scale ageing can significantly lessen the time and costs required. The latter can be achieved by narrowing or screening the field of acceptable candidate materials that would go into long-term qualification tests and thus provide information on the remaining service life of existing structures. Furthermore, improved standards for product and material development are established by defining the performance and behaviour of materials under environmental degradation [7]. 

### 2.3. Ageing Failure Modes in Thermoset Matrices and Composites

One of the most common moisture-induced degradation forms in polymers and GFRPs is plasticisation, which is often a reversible form of degradation. It is associated with an increase in the mobility of polymer chains and may lead to macroscopic swelling and softening of the matrix, reductions in Tg and stiffness, and decreased stress transfer ability of the fibre-matrix interface [6,8,12,15,16]. Plasticisation and swelling are also known to increase the capillarity of moisture in GFRPs [17]. The main degradation effects induced by swelling are a reduction in mechanical and tensile strength of the matrix, the generation of localised stress, cracking and crazing, as well as increased capillarity [9,18,19,20]. 

Moisture ingress in GFRPs can also induce hydrolysis, which is a chemical decomposition process occurring mainly at elevated temperatures. During hydrolysis, a water molecule ruptures one or more chemical bonds. Consequently, both the molecule and the bond(s) compound are separated and a hydrogen and hydroxyl group are formed. In contrast to plasticisation, hydrolysis may cause permanent degradation, since it affects the bonds in the polymer matrix and the fibre-matrix interface [6,7,8,9,12]. In fact, hydrolysis can result in reduced stress transfer in the composite interface and affect all matrix-dominated properties including Tg, off-axis tensile strength, compressive strength, and ILSS [8,21,22,23,24,25].

Hydrolysis is primarily associated with matrix decomposition and leaching [22,23]. Leaching involves the irreversible extraction of low molecular weight, organic additives of the matrix, such as fillers, catalysts, hardeners and pigments, into the ageing medium. The thermal decomposition of the matrix can be followed by its stiffening as well as reduced adhesion and (or) failure of the fibre-matrix interface [2,17,24]. Incompletely polymerised or void-containing and cracked matrices have been found to be particularly sensitive to leaching effects, which can lead to cyclic and long-term, irreversible degradation [3,6,10,12,21,22,23,24,25]. The rate of hydrolytic degradation and hence leaching are primarily governed by the temperature of the ageing environment as well as the thermal properties of the polymer. Increased temperature has been described as an accelerator of moisture-induced degradation. When the temperature of the exposure medium (i.e., water) is higher than the polymer Tg and the material is in a rubbery state, the molecular mobility is increased, which enables an easier passage of moisture through the polymer. Conversely, when the temperature of the exposure medium is lower than the Tg of the material and the material is in a glassy state, molecules “freeze” and thus the diffusion rate is lower [3,7,12]. 

Generally, matrix degradation and interface debonding may in turn change the diffusion kinetics of the material and result in anomalous moisture uptake rates and equilibrium levels. The rate of water absorption is likely to increase once polymer properties have been compromised and degradation has started to take place [7,18,26,27]. It has been observed that exposure to aqueous environments with a higher salinity content results in reduced absorption due to the ability of salt crystals to inhibit moisture diffusion [24]. However, high salinity aqueous media can still induce hydrolytic degradation and impair the properties of thermoset matrices, as well as fibre-matrix adhesion [24,28,29].

As previously discussed, thermosets are often unable to polymerise fully even at high cure temperatures and unreacted polymer sites and curing agent molecules may still be present in post-cured polymer matrices. These are called volatiles and are known as matrix plasticisers. Volatiles exhibit a hydrophilic profile and can be leached out during hydrothermal ageing [9]. Reactions between volatiles and permeated water create stresses both in the matrix and in the interface of GFRPs. In combination with chemical hydrolytic attack, these stresses may generate swelling and osmotic effects, and therefore cracking, crazing, and blistering [9,18,19,20]. Hydrolytic and leaching effects in partly polymerised polymeric materials have been associated with secondary cross-linking. Secondary cross-linking can be classified as chemical ageing since it induces irreversible changes in the matrix network [11,30]. Secondary cross-linking through ageing can be induced for polymeric materials that still possess unreacted polymer molecules. Physical and chemical ageing can act simultaneously. Moreover, the synergistic chemical ageing effects of cross-linking and leaching have been previously observed by several workers. Leaching followed by matrix embrittlement and a subsequent Tg increase can be referred to as “anti-plasticisation”. These effects can significantly affect the final properties of the polymer [10,11,30]. 

## 3. Ageing of Vinyl Ester Matrices and Vinyl Ester-Based Composites

### 3.1. Changes in Polymer and Composite Performance

During their service life, VE-based composites may be exposed to a variety of hydrothermal conditions and it is therefore essential to have a strong understanding of their response in such environments. However, the number of studies on the environmental exposure of vinyl esters and VE-based composites is limited and thus a detailed understanding of their durability and life expectancy is lacking [3,10,31,32,33]. The vast majority of the literature cited in this review focusses on the use of E-glass fibres as the reinforcement in VE-based composites. The properties of E-glass fibres have been extensively reviewed by Thomason and Hartmann and Li and Watson [34,35]. Ghorbel and Valentin [11] reported on the effects of hydrothermal treatment on the physico-chemical properties of neat VE and polyester matrices, as well as VE-based and polyester-based GFRPs. Both neat matrices followed Fickian diffusion during ageing in water at 60 °C, while the diffusion of both GFRPs could be described by the Langmuir model. In disagreement with previously recorded literature results, the VE and VE-based GFRP had higher moisture gains than the polyester and polyester-based GFRP. This was attributed to an incomplete cure of the vinyl ester, as opposed to the polyester, validated by studying the residual exothermic reaction using differential scanning calorimetry (DSC). Plasticisation effects were present, which induced secondary cross-linking for the neat vinyl ester matrix manifested by the decrease in the exothermic reaction in DSC, but without Tg being affected. The polyester-based GFRP exhibited a Tg reduction during ageing, attributed to synergistic hydrolysis and plasticisation. Degradation was also apparent for the vinyl ester-based GFRP in the forms of leaching, cracking, and debonding at the interface. Secondary cross-linking was observed for the VE-based GFRP upon long-term ageing, marked by a Tg increase, although the Tg was initially reduced. The effect was attributed to plasticisation, hydrolysis, and post-curing acting in combination and this was supported by DMA results. Moisture-induced degradation in the FGRPs resulted in higher sorption than in neat matrices.

Wu et al. [36] studied how the use of a number of different silane coupling agents resulted in the formation and structure of an interphase, which altered the hydrothermal performance of unidirectional glass-fibre-reinforced polyester composites. Hot-wet ageing of the compo-sites was carried out by immersion for 72 h in boiling water. The level of fibre-matrix interfacial adhesion was measured using a microindentation technique. The authors concluded that compatibility of the silane tail group with the polymer matrix is more important than chemisorption of the silane onto the glass fibre surface for dry composite strength. However, for hot-wet durability, chemisorption of coupling agent to the fibre surface is critical, and in this case, the compatibility with the polymer matrix has only a secondary effect. Degradation of the interphase during hydrothermal ageing was found to be the primary cause of the reduction in composite flexural strength.

Buck et al. [37] investigated the role of flaws in GF-VE laminates exposed to various environmental conditions while under load in reducing laminate mechanical performance and changing their microstructural properties. Composite coupons with a single edge flaw were hydrothermally aged for up to 330 days in water at 21 °C and 57 °C, with and without a continuous applied load. The effect of the introduction of flaws on the tensile properties and changes in damage mechanisms were recorded. It was found that, under such conditions, the presence of the flaw (edge notch) resulted in more rapid degradation of composite mechanical properties. It was also noted that moisture uptake in the notched coupons did not typically follow a Fickian trend, especially for samples subjected to elevated temperatures or a sustained load. In a further paper, these authors also assessed composite durability by monitoring the Ultimate Tensile Strength (UTS) of similar GF-VE laminates aged under similar environmental conditioning and sustained loading [38]. Sustained loading during ageing gave rise to degradation, resulting in lower UTS and strain-to-failure values. Synergistic ageing and loading effects notably decreased UTS, even upon short-term exposure. Degradation effects were amplified at higher temperatures. More specifically, in the temperature range of 38–49 °C, the UTS of conditioned samples decreased abruptly. At 66 °C, degradation was amplified regardless of loading. Moreover, morphology changes became evident during ageing. Water ageing resulted in the removal of the vacuum side resin layer and left fibres exposed to the ageing medium. Samples aged at room temperature had a similar appearance independent of loading, while greater morphology changes were induced with an increase in temperature and ageing time. Creep effects and sample elongation were observed, which increased with loading and exposure temperature. Matrix cracking and fibre pitting effects were identified, which resulted in debonding at the interface and delaminations.

Chin et al. [26] conducted a characterisation of the sorption and transport of distilled water, salt solution, and a simulated concrete pore solution (23 °C and 60 °C) in films of vinyl ester, isophthalic polyester (isopolyester), and epoxy resins. It was observed that all matrices followed Fickian diffusion under all ageing conditions. Sample weight increase was found to be rapid in the first 10 h of ageing but decayed between 10 h and 100 h, after which equilibrium was attained. Epoxy proved to be the most hydrophilic matrix of the three. The authors attributed this to the high polarity of epoxies compared to vinyl esters and isopolyesters. As expected, the increase in temperature resulted in higher moisture uptake levels and moisture uptake rates. Equilibrium levels did not show a correlation with the polymer polarity measured by contact-angle measurements. This was attributed to the fact that the polarity values obtained were related to the surface and near-surface of the films, and not the bulk polymer. Moreover, the authors noted that microstructures and cross-link density may have influenced uptake kinetics. Surprisingly, isopolyester films aged in salt and pore solution exhibited lower weight increases at elevated temperatures due to leaching upon equilibrium. It is noteworthy that there was no indication of hydrolytic behaviour in vinyl ester matrices, even at elevated temperature ageing.

Zhong and Zhou reported on the effects of accelerated thermal and hydrothermal ageing (both at 95 °C and 150 °C) on the performance of S2-glass fibre-VE composites [2]. Effects of temperature, moisture, and exposure duration on some mechanical properties and microstructures of the composites were investigated, and thermal and hydrothermal degradation mechanisms were analyzed. It was found that the combination of moisture and elevated temperature significantly deteriorated some composite mechanical properties and the property changes were more significant during the early stages of the hygrothermal exposure process. The influence of physical degradation induced by water was found to be less significant than that of chemical degradation resulting from resin decomposition. Optical micrographs of composite fracture surfaces were interpreted as indicating that the matrix resin became fragmented and lost adhesion with fibres in the hydrothermally exposed composites.

Boinard et al. [18] reported on the influence of resin chemistry on water uptake and environmental ageing effects in polyester and vinyl ester GFRP laminates. DMA characterisation of the polyester and vinyl ester laminates revealed an incomplete cure. Anomalies in the moisture sorption were observed for both laminates and neither followed Fickian behaviour. For the polyester laminate, an increase in the temperature of the water medium resulted in amplified degradation. Higher degradation rates were also apparent for water ageing followed by ageing in air. Leaching in water was observed, subsequently followed by anti-plasticisation and stiffening of the material. DMA revealed the weakening of the interface attributed to either debonding or fibre sizing loss. For vinyl ester laminates DMA revealed that unreacted monomer sites plasticised the polymer, through anti-plasticisation effects. The unreacted monomer was leached out during water ageing creating gaps within the FRP network, which allowed excess moisture ingress. However, the ageing effect was found to be reversible, as validated after drying the samples. It was concluded that the vinyl ester laminate exhibited a less hydrophilic nature and was more resilient to moisture-induced degradation than the polyester laminate.

McBagonluri [39,40] carried out a characterisation of fatigue and combined ageing environments on the durability performance of vinyl ester GFRP composites. Cyclic and non-cyclic ageing environments of fresh-water and saltwater from room temperature to 65 °C were employed. No major differences between water and salt water were seen in quasi-static laminate performance following moisture ageing and cycling. In saltwater, reductions of 11% and 32% and an increase of 6% were evident for the quasi-static modulus, UTS and Poisson ratio, respectively. Similar values were obtained for fresh-water ageing, except for the Poisson ratio, which remained unchanged from that of the dry specimen. Cyclic moisture results showed that, although quasi-static material properties were notably reduced after the first cycle, no further reductions were observed from the additional cycles. However, the addition of ageing cycles introduced irreversible ageing affecting both the matrix and the interface. A partial recovery in strength values was only apparent upon re-drying specimens after the first cycle and complete irreversibility was introduced upon the addition of extra cycles. Scanning Electron Microscopy (SEM) indicated that fresh-water ageing promoted cracking around the interface. Fatigue tests indicated that failure occurred due to degradation of the load-bearing fibres and, in the short-term, was independent of the ageing environment, the test parameters, and the nature of the matrix and interface. However, long-term ageing induced matrix toughening and plasticisation, which significantly affected the fatigue performance of the material. 

Research on the response of FRPs conditioned under the synergistic action of moisture and freeze-thaw temperature cycles is an important subject when considering ageing. At low temperatures, the matrix undergoes shrinkage and thermal contraction stress is present at the fibre-matrix interface causing an increase in residual stresses which may result in increased micro-crack formation and debonding of the fibre-matrix interface. Furthermore, any moisture absorbed through capillary effects and subsequently stored in voids may turn to ice at low temperatures. This can result in increased local stresses and can lead to matrix embrittlement, cracking, and even fracture [12]. Karbhari et al. [41] investigated the flexural and impact properties of E-glass fibre-VE at low temperatures. The authors highlighted the suitability of vinyl ester matrices for use in marine and cold-region applications due to their exceptional chemical properties, resistance to osmotic blistering, and high impact resistance. It was observed that the substitution of the reinforcement from a continuous strand mat to a woven layer could potentially increase the damage resistance of the composite. However, this was only the case when the woven surface was placed on top and towards the impact point. Water-induced plasticisation was reported through the formation of hydrogen bonds. These generated bridging within the preform architecture damaged by impact resulting in increased strength on certain occasions. It was found that under certain conditions VE-based composites improve in impact strength and flexural strength at temperatures of −18 °C more than those at room temperature. Immersion in water followed by freezing resulted in the highest moisture absorption, three times higher than room temperature water absorption. Freezing water and room temperature water immersion both resulted in cracking, which facilitated further moisture absorption. In another paper, Karbhari et al. [33] studied the response of ambient cure GF-VE composites after short-term exposure to freezing (−10 °C) and freeze-thaw (−10 to 22.5 °C) conditions, both with and without immersion in water. At low temperatures, the authors observed matrix stiffening followed by a minor increase in the tensile strength and stiffness of the composite. Nevertheless, freeze-thaw ageing compromised the overall performance of the composite. The introduction of water as an ageing agent to the freeze-thaw environment resulted in pronounced degradation of the composite in the forms of matrix hydrolysis and fibre-matrix debonding.

The influence of the molecular weight of glass fibre sizings on the hydrothermal stability of the fibre-matrix interphase in GF-VE composites was investigated by Gorowara [42,43]. Model glass fibre sizings were prepared using formulas taken from patents published by glass fibre producers. These were used as models for the sizings in commercial glass fibre products. The hydrothermal stability of the interphase was characterized by the composite interlaminar shear strength (ILSS), measured before and after hydrothermal ageing. The use of a high molecular weight polyester film former resulted in composites with a 25% greater water uptake. Composites made using fibres sized with a non-reactive silane had a water uptake three times higher than when a low molecular weight film former or no film former was used in the glass fibre sizing. The results showed a clear correlation between the composite hydrothermal durability as measured using ILSS and the equilibrium water absorption levels of composites made using fibres sized with different silanes. 

Karbhari and Zhang [3] reported on the behaviour of vinyl ester GFRP composites with various lay-up designations in aqueous environments. Specimens were aged by full immersion in deionized water at 23 °C and 60 °C. The increase in temperature increased the water uptake rate and the equilibrium level. Moisture-induced degradation was evident in the forms of micro-cracking, discolouration and transverse crack formation generated by micro-crack coalescence, interfacial debonding, and morphology changes. All the aforementioned effects increased the rate of water uptake further. Absorption was highest for the triaxially reinforced specimens and lowest for uniaxial specimens. Nonetheless, all lay-up orientations exhibited a two-stage diffusion. This was attributed either to changes in the viscoelastic response marked by a change in boundary conditions or the following combined effects: relaxation of elastic forces induced by the cross-linked network after the initial weight-gain plateau and degradation at the interface and (or) fibres, enabling wicking, resulting in further moisture gain. At 60 °C, tensile strength and interlaminar shear strength (ILSS) decreased notably with ageing time. Fibre pitting through fibre decomposition (loss of K_2_O and Na_2_O) was noted. This was detected through optical microscopy through the composite structure and was validated by FTIR on leached components. Fibre degradation was attributed to interfacial debonding and cracking. At 23 °C, secondary cross-linking in the matrix was observed after long-term immersion and although tensile strength was initially reduced, it then followed a notable increase reaching values higher than those of the original un-aged composites. In all ageing environments, variations were apparent in tensile strength values. Finally, a Tg increase was observed upon short-term ageing but Tg reductions were observed after the full ageing period of 52 weeks.

Fraga et al. [44] studied the effects of water absorption in unsaturated isophaltic polyester and vinyl ester matrices and their GFRP composites. The viscoelastic response of the materials was assessed by DMA after re-drying the specimens. Interfacial adhesion was studied by ILSS testing and SEM. DMA results of the dry vinyl ester matrix and composite demonstrated a double peak in the tan *δ*, which the authors attributed to thermal instability. At 40 °C, Fickian behaviour was displayed by the vinyl ester matrix, with most water being absorbed during the first 140 h of immersion. No equilibrium was reached for the polyester samples, which exhibited a continuously increasing weight gain pattern and a significantly higher moisture gain. This effect was attributed to a high composite void content and the high polar-hydrogen donors in the hydrogen bonding-carboxylic chain of polyester samples making them more hydrophilic. The void content of both matrices increased with ageing, although polyester was more susceptible to the effect. Moreover, the polyester matrix exhibited a notably lower Tg (78 °C) than vinyl ester (110 °C) contributing further to the hydrophilicity of the former. The weight gain of the polyester matrix was also higher than the vinyl ester at 80 °C; nonetheless, after a maximum weight gain had been reached for the former matrix, an abrupt decrease to negative values followed. The effect was attributed to the leaching of unreacted monomers or oligomers. When a higher styrene content was introduced to the polyester matrix, its hydrophobicity increased. Resins with a lower styrene concentration exhibited the easier extraction of residual unreacted components [44].

The composites treated at 40 °C featured similar weight change trends to the neat matrices; nevertheless, both composites attained similar saturation levels to each other. The water uptake of the polyester composite was higher than expected, due to interfacial degradation and cracking of the matrix. At 80 °C, a weight gain maximum was attained by both composites, which was then followed by abrupt weight decreases, eventually dropping to negative values. This effect was greater for the vinyl ester composite. The authors attributed the effect to a combination of hydrolytic degradation of the matrix and the silane coupling agent on the fibre. The latter is known to be activated upon immersion in water between 50 °C and 100 °C. Poor interfacial adhesion was identified by SEM in both aged and un-aged specimens, attributed to a non-compatibility of the fibre sizing. At 40 °C, the matrix of the polyester composites exhibited a brittle nature, whereas swelling was induced at 80 °C resulting in a primarily ductile matrix. The matrix of the vinyl ester composite was found to be brittle at both ageing temperatures. Although ILSS values were found to be higher for the vinyl ester composite, the fractional strength retention upon ageing was similar for both composites. Secondary-cross-linking through anti-plasticisation was observed for both matrices, manifested by increases in Tg and cross-link density (decrease in DMA tan *δ*). Similar behaviour was exhibited by the composites [44]. 

Hammami and Al-Ghuilani [45] examined the durability of vinyl ester GFRP composites in seawater and corrosive fluids in, hot, humid, or dry conditions for 3 and 6 months. A high composite fibre content resulted in poor wetting and therefore reduced interface adhesion, while it also promoted micro-crack formation and layer delamination. Such failure mechanisms can accelerate water diffusion through the composite structure and thus degradation. For seawater ageing, a decrease in flexural modulus of 18.5% was recorded between 3 and 6 months of ageing. An initial linear water uptake trend of 1–1.5% gain was observed between 25 and 100 days, respectively, followed by a secondary plateau of approximately 2.5% gain after approximately 185 days. Matrix leaching was reported, which resulted in reduced interface adhesion leaving fibres without protection. Moreover, water ingress resulted in volumetric expansion and, in turn, in the formation of micro-cracks. Similar observations but with increased degradation effects were recorded in hot (exceeding 50 °C) and humid environments. Six months of exposure to a hot (up to 60 °C) and dry environment resulted in secondary cross-linking and an increase in flexural modulus. An increase in corrosive fluid concentration and (or) ageing time resulted in increased degradation for the GFRP. Degradation was observed in the form of corrosive behaviour, including hydrolytic and oxidative corrosion as well as pit formation and blistering. Decreased ILSS values, attributed to the presence of micro-cracks, were found for composites exposed to both humid and acid-rich environments. Conversely, an increase in ILSS values was observed after exposure to hot and dry environments. 

Karbhari and Wang [30] conducted a multi-frequency dynamic mechanical thermal analysis of moisture uptake in vinyl ester GFRP laminates. The residual cure of the laminates was accelerated by the presence of moisture. More specifically, ambient exposure of GFRPs led to a significant Tg increase after 6 months. During that period, a Tg increase was observed for ageing in DI water and pH 4 buffer solutions. On the contrary, Tg decreased for pH 7 buffer and 24 h ‘wet–dry’ cycling conditions, while fluctuations in Tg were seen for pH 10 buffer solution immersion. When trying to understand these Tg changes, the competing effects of secondary cross-linking and environmental attack were considered. Water-induced degradation was present in the forms of leaching and anti-plasticisation. Moreover, the authors reported that DMA testing of the GFRP in the longitudinal direction produced higher Tg values than testing in the transverse direction. Lastly, an increase in the DMA test frequency resulted in an increase in the observed Tg value. 

**Figure 1:**
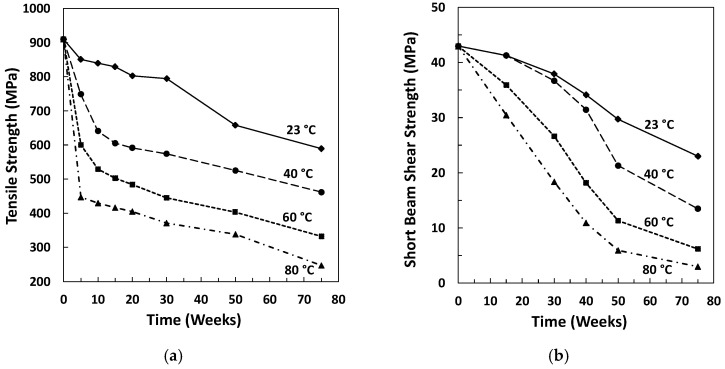
Effect of hydrothermal ageing in deionized water at different temperatures on the mechanical properties of pultruded glass fibre-reinforced vinyl ester composites (**a**) Tensile strength; (**b**) Short beam shear strength. Reprinted/adapted with permission from Ref. [46]. 2004, Elsevier Ltd.

Chu and Karbhari [10,46] studied the effect of water sorption on the performance of pultruded vinyl ester GFRP composites aged by full immersion in DI water at 23, 40, 60, and 80 °C. Aged specimens exhibited Fickian behaviour at all conditions and the rate of water uptake rate and equilibrium weight gain both increased with temperature. With high-temperature ageing, wicking along the interface and micro-cracks of the composites, and thus moisture storage were observed, increasing the total gain to values higher than the theoretical values. A change in the colour of the specimens, indicative of hydrolysis, was also observed. Short-term ageing of 5 weeks led to a small decrease in Tg. However, after 10–15 weeks a Tg increase was noted along with a decrease in storage modulus. The storage modulus decrease was attributed to hydrolysis, which was confirmed by a preliminary FTIR analysis. Higher ageing temperatures resulted in an overall lower decline in glass transition temperatures. This was attributed to leaching effects, which led to matrix embrittlement, followed by chain scission. Nevertheless, the effect was not apparent with low-temperature ageing. At 80 °C, fibre pitting, cracking and weakening of the interface were confirmed. In the first 5–10 weeks of ageing, most of the loss in tensile strength could be recovered upon re-drying the laminate. The reduction was attributed to plasticisation effects, which, as previously discussed, are mostly reversible. At 23 °C exposure, ILSS loss was found to be linear with ageing time. At 40 °C, a stepped reduction was observed—mostly irreversible damage—attributed to failure at the interface in the forms of debonding and osmotic cracking. At higher temperatures, leaching, debonding, and micro-crack coalescence to form transverse cracks, and therefore higher moisture uptake and increased degradation, were observed. The levels of the tensile strength (Figure 1a) and ILSS (Figure 1b) retention decreased with ageing time and conditioning temperature.

Yu et al. [47] reported on the hydrothermal ageing of pultruded vinyl ester resin CFRP composites. Specimens were exposed to distilled water and brine (3% NaCl) at a temperature range of 65–95 °C. Fickian diffusion plateaus were observed at all conditions with increased uptake rates observed as a function of temperature. Although the fibre-matrix interface was more resilient during absorption due to the hydrophobicity of the reinforcement, water-induced plasticisation of the matrix after long-term ageing was reported resulting in two distinctive Tg values. The authors reported that long-term moisture ageing induced secondary cross-linking and a subsequent rise in Tg. However, the effects of dual Tg appearance and cross-linking were reversible upon re-drying of the specimens. SEM imaging showed debonding at the interface after long-term exposure, which in turn contributed to reduced flexural strength and ILSS. Lastly, no major difference was observed between the distilled water and salt solutions. 

Sobrinho et al. [22] reported on the effects of water absorption on an epoxy-based vinyl ester polymer. Fickian-like behaviour was observed in the first stages of sorption followed by small but abrupt weight increases. Thereafter, a subsequent weight drop was reported, whereby leaching dominated sorption. After 16 days of ageing in water at 60 °C, discolouration and voids were identified in the samples, which intensified with ageing time. Mechanical testing and thermal analysis results suggested matrix embrittlement through secondary cross-linking of the matrix in the hydrothermal medium. Long-term ageing resulted in decreased Tg and Young’s modulus and an increase in strain and fracture toughness—this after the latter two properties had undergone an initial decrease upon short-term ageing. It is noteworthy that samples used for this ageing study were only cured at room temperature but were compared to un-aged samples post-cured at 120 °C. As previously discussed, the post-cure step is highly important for the performance of vinyl esters, while incompletely cured vinyl esters are more susceptible to hydrolytic degradation [45,48].

Narasimha Murthy et al. [49] investigated the seawater durability of epoxy and vinyl ester GFRP, and also epoxy and vinyl ester CFRP composites. All aged composites exhibited a Fickian-like weight gain pattern. Seawater resulted in expansion and shrinkage and in-turn fibre/matrix debonding, cracking, and voids, which functioned as water storage regions and increased total moisture gain. Overall, VE-based composites were found to be less hydrophilic than epoxy-based composites, exhibiting lower weight gains. As a result, VE-based composites featured greater flexural strength, ILSS, and tensile strength retention than epoxy-based specimens. All mechanical properties underwent notable degradation during water uptake prior to equilibrium. 

Visco et al. [50] presented a comparative study on the effects of hydrothermal seawater ageing at 60 °C in an isopolyester and a vinyl ester polymer. Vinyl ester was found to be more hydrophobic and less susceptible to seawater degradation than the isopolyester. Thus, the vinyl ester polymer retained better mechanical and physical behaviour than the isopolyester. Fickian trends were observed for both polymers. A change in specimen colour was detected upon long-term ageing indicative of hydrolysis, along with secondary cross-linking, which is in line with the observations of Chu and Karbhari [10]. Progression of ageing-induced void formation and thus further degradation of the isopolyester was reported. In vinyl ester, degradation was primarily present on the outer surface of the resin; detached surface layers caused by hydrolytic attack were apparent. This observation was in accordance with the findings of Buck et al. [40], who detected the removal of the outer resin layer of vinyl ester GFRPs upon hydrothermal ageing. Visco et al. highlighted that the structural features of a matrix need to be considered for its potential use as a laminate constituent. These can dictate the stability of polyester and vinyl laminates in seawater, especially when taking into account the different chemical reactivity of the ester groups of the two resins. In line with the observations of Ghorbel and Valentin [11], it was found that the moisture uptake kinetics of the polymers were strongly dependent on their degree of cure. 

Eslami et al. [29] reported on the effects of moisture absorption on the degradation of vinyl ester GFRP composite pipes and the modelling of transient moisture diffusion using finite element analysis (FEA). Fickian behaviour was exhibited by all aged specimens at all conditions. Composites aged in seawater reached equilibrium faster but exhibited lower diffusivity and moisture gain levels than composites aged in water. In buckling tests, the failure force and displacement were reduced with an increase in the temperature of the water and when the exposure medium changed from seawater to water. Moreover, SEM confirmed a weakening at the interface with an increase in ageing temperature. Finally, the authors claimed that FEA could successfully predict moisture diffusion rates and equilibrium values for the aged specimens.

Sarlin et al. [20] conducted a comparison on the ageing of vinyl ester GFRP pipes made using bisphenol-A-based and novolac-based vinyl ester matrices. Water immersion was found to induce higher levels of degradation, in the forms of leaching and weakening of the interface, than corrosive solution. Novolac-based laminates had a higher weight gain, which nonetheless was unexpected since novolac-based vinyl esters are known to be more hydrophobic than bisphenol-A-based vinyl esters. Water immersion resulted in the greatest weight gain of all ageing media. The second largest weight gain was obtained in 5% H_2_SO_4_ medium, while the lowest weight gain was noted in the environmental chamber (70 °C, 95% RH). Subsequently, the greatest tensile strength reduction of up to 65% was observed in the water medium and the lowest tensile strength reduction of 10–25% after 12 months was shown in the environmental chamber. Although one of the novolac-based GFRPs had the highest weight gain in all ageing media, this was not correlated with the tensile strength loss of the composite, which achieved the highest long-term tensile strength retention. The highest tensile strength reduction was exhibited by one of the bisphenol-A-based GFRPs. Moreover, the authors confirmed that a reduction in tensile strength is not necessarily correlated with changes in GFRP stiffness; for instance, one of the bisphenol-A-based samples underwent the highest tensile strength reduction, but its stiffness remained unaffected. 

Yin et al. [51] reported on the weight changes, hydrothermal expansion, and thermomechanical properties of a vinyl ester matrix intended for GFRP applications when immersed in water or an alkaline solution. For water immersion, the total weight gain increased with an increase in the temperature of the ageing medium. However, the opposite effect was observed in the alkaline solution, although the decrease was not considered significant. According to the authors, weight change levels were affected by water-induced relaxation and leaching. For water immersion, matrix relaxation was the dominant effect over leaching, resulting in an increase in weight gain. In both ageing media, diffusivity values increased with an increase in ageing temperature. The alkaline medium induced higher hydrolytic rates than water, resulting in higher mass loss. Tg reductions attributed to plasticisation effects were observed in both ageing media. This effect was reversible and leaching was considered to occur only on the specimen surface. Lastly, an increase in the polymer tensile strength and elongation at break was observed after 6 months of ageing.

The changes in the performance of pultruded GFRP composites with either vinyl ester or unsaturated polyester (UP) matrices was investigated by Sousa et al. [52]. Hydrothermal ageing experiments were conducted by immersion in demineralised water and salt water at 20 °C, 40 °C, and 60 °C. The effect of a 100% RH atmosphere at 40 °C was also included in their study. Composite ageing was carried out for up to 720 days. A variety of performance parameters of the aged composites were assessed after further desorption conditioning until a constant weight was reached. Moisture uptake under the different ageing conditions showed an approximately Fickian response. In comparison to the VE-based composites, the UP samples exhibited a higher moisture uptake under almost all of the different ageing environments studied. It was found that the VE-based composites had overall better mechanical performance than the UP composites. After 720 days of ageing, the highest reductions in composite tensile strength (−45% and −33% for the UP and VE composites respectively) were caused by water immersion at 60 °C. The same authors have also published a paper presenting the results of long-term (up to 100 years) prediction models for the mechanical property retention of their pultruded GFRP materials [53]. The predicted performance retention values indicate a higher overall degradation level in demineralised water immersion compared to salt water immersion. They stated that the predictions of their model exhibited a higher variation in the prediction of performance retention compared to other available models in the literature.

### 3.2. Micromechanical Characterisation of the Interface in Hydrothermally Aged Composites

As previously mentioned, it is well known that the stress transfer capability of the fibre-matrix interface region plays a critical role in defining the durability of GFRP materials and their associated structures over their lifetime [2,6,8,12]. Moisture penetration at and along the interphase region between the matrix and the glass fibre has frequently been proposed as a probable path for the acceleration of moisture infiltration into GFRP. Absorbed moisture has been shown to cause the hydrolysis of the interfacial bonds, which can significantly reduce the load transfer capability between the matrix and the glass fibre [6,8]. Nevertheless, detailed knowledge of the effectiveness of studying interface performance in wet environments as a means of accelerated ageing and how to accurately determine the hydrothermal response of a bulk laminate is still somewhat limited. Micromechanical single fibre composite testing of the interfacial shear strength (IFSS) is one of the few available experimental routes to directly probe the stress transfer capability of the fibre-matrix interface region. It has been shown that changes registered at the microscale often correlate with changes in composite macroscale properties [6]. The number of published studies of this type of direct micromechanical investigation of the hydrothermal ageing of the fibre-interface in vinyl ester composites is limited. Moreover, until recently, the effect on the interface was mainly extrapolated from composite performance in tests such as the short beam shear test [3,42,43,44,45,46,47,48,49,54,55].

A significant volume of data regarding the micromechanical single-fibre testing of various fibres with epoxy resins and with many different thermoplastic polymers can be found in the literature. The literature on the micromechanical single-fibre testing of the composite interface is heavily weighted towards the study of epoxy resins and thermoplastic polymer matrices. Reports on microbond testing using polyesters, and in particular, vinyl esters, are less common [9,56,57,58]. This shortage of literature may in part be due to the challenges of producing acceptable VE-based samples for micromechanical testing. 

Ash et al. reported on the use of the microbond test to study the IFSS of polyester on bare and silane-coated glass fibres. They noted significant sample preparation issues and attributed incomplete polyester microbond sample curing and a related scatter in the experimental results to the evaporation of up to 60% of the styrene in the resin out of the microbond test droplets during sample preparation and curing [56]. Laurikainen [9] and Dirand et al. [57] have also proposed a similar hypothesis relating to the evaporation of styrene from vinyl ester microbond sample droplets. Both papers report that significant problems were experienced during microdroplet sample preparation using the very scale resin volumes required, and this was exhibited in the poor curing of the polymer resins. Results of the investigation on the curing behaviour of their VE resin combined with previous results reported on microdroplet testing of a similar VE resin led to a hypothesis proposing that the cause of the problem was vaporisation of the styrene from the resin droplets [57]. Laurikainen claimed that this effect could be counteracted by curing microdroplet samples in a styrene-rich atmosphere in a closed container [9]. More recently, Bénéthuilière et al. also reported using this styrene-rich curing atmosphere method to successfully prepare VE resin microdroplets [58]. They conducted an ageing study (95% RH at 70 °C) of a conventional styrene-based and a styrene-free vinyl ester resin, assessed by the microbond test. The IFSS of glass fibre and the dry styrene-based vinyl ester was approximately 40 MPa. An IFSS loss of 22% was recorded for the styrene-based matrix, while no IFSS loss was apparent for the styrene-free matrix. The authors attributed the effect to hydrolysis through bond rupture of polyester pre-polymer found in the styrene-based matrix. These results were in disagreement with the ageing results obtained on bulk-scale UD laminates. Cracks and porosity induced by ageing were featured in styrene-poor composites in the bulk scale, attributed to the hydrolysis of the 1,4-butanediol dimethacrylate reactive solvent. Nonetheless, no hydrolytic effects were observed for the styrene-based composites.

Xypolias et al. and Thomason et al. have reported on the use of the microbond test to investigate the IFSS in various glass fibre-vinyl ester (VE) composite systems and a number of issues related to the sample preparation were identified [59,60]. Cure schedules that produce well-reacted VE polymers on the macroscale did not result in cured microdroplets. Hence, the microbond test could not be carried out on samples with the same cure history as macroscale composites. Testable microdroplet samples could only be obtained when resin cure was carried out under an inert atmosphere. Higher IFSS values were obtained by raising the final temperature of the cure schedule. Glass fibres with a full sizing gave significantly higher apparent IFSS values compared to bare fibres or fibre coated with only silane coupling agent. It was discovered that the measured IFSS of VE-compatible glass fibres was approximately doubled when fibres were mounted using epoxy glue instead of cyanoacrylate glue. They concluded that great care must be taken in ensuring that effects observed using the microbond test are evidence of real material characteristics and not artefacts of sample preparation. In another paper, Thomason and Xypolias reported the results of a study of hydrothermal ageing on the apparent IFSS of GF-VE microbond samples [54]. The scale related issues of estimating water absorption levels in microbond samples were reviewed and discussed and a solution based on Fickian diffusion was presented. The IFSS of microdroplets was shown to be relatively stable for up to 6 days of ageing in water at 23 °C. However, longer exposure resulted in rapid degradation in the interface strength. Immersion in water at 50 °C resulted in an immediate 20–30% irreversible loss in strength. Further exposure beyond two days in water at 50 °C led to a total loss of integrity of the microbond samples.

The single-fibre fragmentation test (SFFT) is another micromechanical test from which a value of IFSS can be derived. An advantage of this method is that, as in a real composite, moisture must first diffuse through the polymer matrix before interaction with the fibre-matrix interface can occur. At the same time, the value of IFSS obtained is also dependent on having accurate and reliable information on the single-fibre strength at very short (sub-millimetre) gauge lengths. Consequently, the results of ageing experiments must take into account changes in the fibre strength as well as changes in the fibre-matrix interface. Cheng et al. reported the use of a fragmentation technique to study the IFSS of water-sized and aminosilane-coated E-glass fibres with vinyl ester and epoxy resins [61]. The glass fibres in this study were aged in both warm (50 °C) and hot (100 °C) deionised water for 24 h or 4 h respectively. The IFSS results were used to demonstrate that molecularly thin layers are effective adhesion promoters in GFRPs. It was suggested that epoxy resins adhere through aminosilane coupling reactions, but for the vinyl ester resin, the maximum adhesion probably occurred through aluminium hydroxide sites on the glass fibre surface.

Sjogrena et al. [62] investigated the effects of two different glass fibre sizings on the IFSS of GF-VE using the single fibre pullout test and the SFFT. A sizing of polyvinylalcohol with no silane coupling agent was used to obtain a weak interfacial interaction (NoCA). whereas a combination of an unsaturated polyester dispersion film with a methacrylsilane coupling agent was employed to obtain a stronger interface (CA). An Owens–Corning commercial glass fibre product was also tested for comparison. The study revealed a limitation with single-fibre composite tests for fibre-matrix combinations with high interfacial toughness. Neither of the two single-fibre tests could be used to quantify the interfacial failure of the CA fibre samples as no interfacial failure was observed. However, cross-ply laminates based on the NoCA fibres demonstrated significantly lower transverse cracking toughness in comparison to laminates containing the CA fibre laminates. The composite laminates based on the commercial glass fibres performed almost as badly as the NoCA fibre-containing laminates. The authors also concluded that the high transverse cracking toughness of the CA material was at least partly due to the strong interface adhesion and the high ductility of the fibre-matrix interphase. 

Fichera et al. investigated the effects of seawater on the transverse tensile strength of a carbon fibre–VE composite investigated by tensile testing of single-fibre composites and also unidirectional composite laminates [63]. The performance of the fibre-matrix interface was probed using the transverse tensile single-fibre (TTSF) test with specimens aged in a seawater tank held at 40 °C until saturation was reached at approximately 0.5% weight increase. They reported that a combination of the low carbon fibre diameter and the severe brittleness of the VE matrix polymer made it difficult to observe the initiation of debond failure in their samples. However, they were able to determine the tensile strength of the fibre-matrix interface through a combination of finite element stress analysis and the value of the debond initiation load, which could be identified in the load–displacement data. Interestingly, it was found that seawater exposure of the TTSF samples had no significant influence on the measured interface strength. 

Ramirez et al. studied the degradation of the mechanical properties of vinyl ester and epoxy matrix composites, containing either glass or carbon fibres, aged in distilled water at room temperature, 40 °C, and 60 °C until saturation [64]. SFFT was used to determine the fibre-matrix interface shear. SFFT results revealed substantial fibre-matrix debonding and significant reductions in the IFSS of all systems after exposure to water. The IFSS retention of the E-glass reinforced composites was better than that of the carbon fibre composites. However, it was shown that the E-glass fibres were also degraded by water exposure, particularly at higher water temperatures. All composite types exhibited significant changes in transverse strength after water immersion. It was proposed that this was related to the degradation of both the matrix and the interface performance caused by water. The authors stated that the single-fibre and composite tests results were supportive of the hypothesis that water degradation of the interface was a major cause of the lower performance of the hydrothermally aged composites. The following year, Ramirez and Carlsson published modifications to the SFFT procedure that they suggested allow the separate identification of the fibre break and fibre-matrix debond propagation events [65]. The method was used to characterize the fibre-matrix interface toughness of E-glass-VE samples before and after saturation in seawater at 40 °C. By focusing on the debond propagation event of the SFFT, and by measuring the critical load for debond propagation, the authors were able to use fracture mechanics analysis to determine a value for the fracture toughness for the interface. They showed that hydrothermal ageing in seawater caused significant degradation of the fibre-matrix interface with the fracture toughness being almost halved after ageing. 

Yang et al. also used SFFT in their investigation of the influence of the fibre-matrix interface of pultruded glass fibre-reinforced polyester composites exposed to hygrothermal environments, including deionized water immersion and saltwater immersion at 30 °C and 60 °C for 180 days [66]. After 180-day immersion at 30 °C, the IFSS determined by SFTT decreased by 21% in deionized water but only 1% in salt water. However, immersion at 60 °C resulted in an IFSS loss of 53% in deionized water but only 23% in salt water. Unfortunately, the authors gave no information on their values for glass fibre strength at fragmentation length, how these were obtained, or whether the values changed with fragmentation sample ageing.

The SFFT analysis in these papers illustrates the challenges of using SFFT for this type of investigation. The estimate of the fibre strength at the fragment gauge length was obtained by the standard method of estimating the Weibull parameters for the fibres using measurements at longer, achievable gauge lengths and then using the Weibull parameters to estimate the fibre strength at much shorter lengths. This is a major weakness of the SFFT method, which requires extrapolation over a large length scale outside of the measured parameters with a very significant error range on the obtained value. The Weibull parameters are also obtained on dry fibres not encased in polymer, a situation known to affect the measured breaking strain of the fibres. Similarly, the authors have estimated the strength of glass fibres in the fragmentation samples using values obtained from fibre exposed to water without the polymer encapsulation, a situation that is much more aggressive for glass fibre degradation. These comments are in no way a criticism of the authors’ excellent experimental procedure but given to once again emphasise the challenges involved in using these micromechanical tests to investigate the ageing of the fibre-matrix interface and to put any conclusions drawn from such testing, particularly when only micromechanical testing is carried out.

### 3.3. The Influence of Voids in the Ageing Behaviour of Composites

It is commonly accepted that it is difficult to manufacture composite materials entirely free of voids, which can significantly compromise the environmental response and mechanical performance of composites [5,67]. In GFRP composites, voids can appear in many sizes and shapes such as air bubbles in the resin matrix or at the fibre-matrix interface. However, matrix cracks and delaminated regions can also act as voids. These can all function as low resistance paths for moisture into the structure of a composite material diffusion [6]. The two main causes for bubble void formation are air entrapment and vaporisation of volatile components or contaminants during cure. Air entrapment usually appears in the initial manufacturing stage of polymeric composites either due to air bubbles being trapped in viscous resin formulations during their preparation or due to incomplete wetting of the filaments or fibre bundles. Poor wetting can be attributed to the high viscosity of resins, which hinders the penetration of the latter into the fibre bundles creating air gaps. A second cause of void formation is particularly related to the high-temperature curing of resins. During high-temperature curing, voids may be formed by the vaporisation of volatile components or contaminants. It has been observed that such voids can significantly lower some mechanical properties of composites [51]. Another common cause for the appearance of voids, cracks, and interface failures in GFRPs is the differential coefficient of thermal expansion between the fibre and the matrix. When accompanied by large temperature fluctuations, this mismatch can result in the generation of residual stresses and, in turn, activate failure mechanisms such as fibre fracture, delamination, and cracks [6,17].

As soon as GFRP has been exposed to a humid environment, moisture starts increasing in the outer surface of the material. It then continues to penetrate the composite structure over time, while cracks tend to grow parallel to the free surface. Crack growth is strongly dependent on the different degrees of loading applied to the material. At low magnitudes of loading, crack growth is primarily governed by chemical reactions. At moderate loading magnitudes, crack growth is mainly controlled by the diffusion process, while at higher levels of loading, cracking is affected by stress-assisted corrosion [6,19]. According to Gagani and Echtermeyer [68,69], cracks located at the external layers of GFRPs, which are in direct contact with the ageing fluid, can accelerate diffusion significantly. Conversely, cracks located within the internal layers of the materials had no significant impact on the diffusion kinetics.

The exposure of GFRPs to aqueous environments has been associated with the activation of osmotic effects. These are initiated immediately after the exposure of the material to water. Once the water has started entering the structure of the material, reactions with its hydrolysable components will start occurring resulting in micro-cavities of concentrated solution in the polymer network. These moisture-containing micro-cavities lead to crack propagation and delamination initiation enabling further moisture diffusion through the material. This effect is commonly known as “osmotic cracking” and is one of the most common failure modes of composites exposed to aqueous environments [12,25].

Delamination is a critical damage mode of thermoset composites, which is often induced by the presence of moisture at the interface of the two adjacent fibre plies. Delaminations in a laminate develop due to excessive out-of-plane or interlaminar stresses produced at the interfaces between adjacent fibre plies [70]. It takes place when the resultant transverse shear force exceeds a threshold value. Similar to cracking, delamination allows the storage of moisture within the laminate and accelerates diffusion [6]. According to Gagani and Echtermeyer [68,69], fluid diffusion in multi-directional laminates was increased by five times by the presence of delaminations. The causes of delamination in a hydrothermally exposed GFRP are usually the same as those of cracking, such as hydrolysis and volumetric expansion, osmosis and blistering, residual stresses, thermal fatigue, and interfacial failures [6,12]. Even though delamination can compromise the life of a laminate through premature buckling, stiffness degradation, and moisture permeation, in certain cases, it may improve stress relief and enhance its properties. More specifically, in short-term moisture ageing, delamination may result in damage growth and premature failure. Conversely, in long-term ageing, delaminated areas may still function as load-bearing layers in a laminate [6]. 

The available data around the effect of voids on the water absorption of polymeric composites are limited, possibly due to the difficulty of manufacturing composite samples with a controlled void content [71]. Furthermore, as previously mentioned, the thermoset composites literature tends to be heavily weighted towards epoxy-based FRPs in many areas and this is also the case for the influence of voids. It has been shown that the effect of voids on the water uptake kinetics of polymeric materials and their performance after water exposure is significant and it should not be neglected since increases in void content can result in large increases in moisture uptake rate and saturation levels [5,71,72,73,74,75,76,77]. With regard to VE-based composites, the Carlsson group has published a number of reports on the effects of voids in VE-based FRPs and the results generally reflect those observed in epoxy composites [78]. Fichera and Carlsson analysed the moisture uptake in unidirectional composites (seawater immersion at 40 °C) using both the standard diffusion model and the capillary flow model, which assumes that voids form channels following each fibre along the panel [79]. All their composite panels exhibited higher moisture saturation levels compared to a neat VE resin—this even though the panels contain 65% volume of carbon fibre, which was assumed not to absorb any moisture. The diffusion model predicted much longer times, and the capillary model much shorter times, to saturation than observed experimentally. The authors noted that the diffusion model is based on the assumption of a perfect fibre-matrix interface while the capillary model assumes the unobstructed capillaries which run the length of the model laminate. They proposed that, in reality, voids at the fibre-matrix interface are probably isolated from each other, and that capillary flow is likely disrupted by areas of good fibre-matrix bonding.

Galpayage Dona et al. also modelled the void-dependent kinetics of water uptake kinetics in similar unidirectional VE-based composites under similar conditions [80]. The model was based on the assumption that two-phase flow could be used to describe the dynamics of capillary filling of voids. It was further assumed that void filling could be modelled using Poiseuille flow and gas permeation through the capillary. The largest proportion of the total water uptake was found to be due to capillary filling, which is rapid during the initial regime and decreases until saturation. A good agreement between experimental and simulation results was found at a contact angle between water and the fibre surface of 29.7° for the capillary (void) radius of 1.2 μm. The authors stated that the results of their study suggest that this type of model may be applied to investigate the dynamics of water uptake in FRP composites that contain voids at the fibre-matrix interface. They also proposed that the model can be further extended to predict what the influence of microcracks and other structural defects may have on composite water absorption.

## 4. Concluding Remarks

- The growing demand for composite materials in infrastructure applications, where exposure to environmental conditions is inevitable, makes ageing studies a necessity. However, the in-service examination of large composite structures can be a time-consuming process involving high costs. Therefore, accelerated ageing conditions are widely employed in laboratory-based composite research. The basic concept of accelerated ageing is the increase in degradation conditions, such as temperature or humidity to a higher level than that achieved in real-life operating environments. Ageing can be reversible (physical ageing) and (or) irreversible (chemical ageing). Chemical ageing primarily involves the combined action of degradation agents, such as moisture and temperature, known as hydrothermal ageing. Consequently, it can be concluded that particular care should be employed in the interpretation and extrapolation of results when accelerated hydrothermal ageing is applied to composite materials since the increase in degradation effects can induce irreversible damage modes in the material, which would not be apparent in a real-life ageing environment.
- Comparative studies showed that vinyl ester matrices can be less susceptible to moisture attack than polyesters, isopolyesters, and epoxies, provided that they are sufficiently cured. When sufficiently cured, diffusion in vinyl esters was primarily observed to follow Fickian trends. However, for incompletely cured or void-containing matrices, and (or) when high-temperature ageing is employed, the diffusion kinetics can vary. Water uptake in vinyl esters and their composites was found to induce two main degradation effects: plasticisation after short-term ageing and hydrolysis after long-term ageing.
- Degradation effects induced by plasticisation are primarily reversible or partly reversible and can affect the mechanical properties, moisture levels and Tg of vinyl ester and VE-based composites. Conversely, hydrolysis is associated with irreversible degradation and chemical changes in the polymeric material. Hydrolysis is governed by leaching, which can, in turn, result in fibre-matrix interface weakening and debonding, mechanical property and Tg depression, cracking and micro-cracking, delaminations, increased levels of moisture uptake, and secondary cross-linking through anti-plasticisation effects. Vinyl ester matrices, and, in turn, their composites, may be particularly sensitive to secondary cross-linking and anti-plasticisation since they often exhibit incomplete cure due to their high degree of heterogeneity and microgel formation during curing.
- Changes in the stress transfer capability of the fibre-matrix interface region during hydrothermal ageing play a critical role in defining the durability of VE-based GFRP materials and their structures during their service life. However, the number of studies using micro-mechanical testing methods for the direct evaluation of the durability of the composite interface is limited, especially regarding the use of a VE matrix. Since the interface of single fibre micro-composites is often different from that of bulk composites, direct property transferability between the two scales upon ageing can be ambiguous. However, encouraging indications have been recorded regarding the understanding of damage modes and evolution, as well as strength retention trends at the interface. Taking into account the benefits and capabilities of micro-mechanical testing, it can be concluded that, if micro-mechanical testing methods are used with caution, they can provide fundamental information on the bulk composite material as a function of environmental history.
- It can also be concluded that more information about the effects of voids in synergy with hydrothermal ageing is required. Voids have been found to increase the performance degradation rate in GFRPs exposed to hydrothermal environments. Even small levels of composite void content are found to result in highly significant changes in moisture uptake behaviour and equilibrium level. Voids, cracks, and delaminations are known to accommodate moisture within polymer networks and thus further increase moisture-induced degradation and crack/void growth and propagation rates. The resultant effects on the mechanical properties of GFRPs, mainly the ones governed by the matrix and interface, have been reported to be critical.

## Data Availability

Not applicable.
